# Motivational interviewing may facilitate professional interactions with inspectees during environmental inspections and enforcement conversations

**DOI:** 10.7717/peerj.508

**Published:** 2014-08-19

**Authors:** Lars Forsberg, Hans Wickström, Håkan Källmén

**Affiliations:** Department of Clinical Neuroscience, Karolinska Institutet, Stockholm, Sweden

**Keywords:** Behaviour change, Intervention, Environmental behaviour, Environmental inspections and enforcement, Motivational interviewing

## Abstract

In the present work inspectors used Motivational Interviewing (MI) to promote environmentally sustainable behaviour in inspectees. MI is a counselling method with scientific support for various health behaviour changes. Inspectors (*n* = 32) in four Swedish municipalities received training in MI over a yearlong period. Their MI competency as well as their experience of using MI in routine inspections was monitored over the year. The results showed that inspectors significantly increased their competence in the Empathy variable, defined as accurate listening to inspectees. Inspectors judged MI to be useful in inspections, approximately 5 on the 6-point scale. There were indications that MI may be easier or more appropriate to use in certain inspections than in others.

## Introduction

It could be said that we are all responsible for taking care of environmental resources in a sustainable way, yet it is hard to know what should be done and by whom. In Sweden, overall responsibility for environmental policy implementation lies with the Swedish Environmental Protection Agency (Naturvårdsverket). This includes ensuring compliance with the Swedish Environmental Code, whose aim is “to promote sustainable environment for present and future generations” (Svensk författningssamling, 1998:808). However, municipalities and county administrative boards are responsible for the implementation of the Environmental Code within their territories.

Each municipality and county administrative board employs environmental protection inspectors who are tasked with fostering good environmental behaviour among inhabitants. The inspectors’ primary task is to implement and achieve compliance with the Environmental Code. Inspectors carry out their role by implementing control measures, and providing information and guidance. Ideally, compliance with the Environmental Code could be reached through self-regulation, which would entail a minimal level of inspection and enforcement costs.

The present study evaluated conversations between environmental inspectors and inspectees, during both pre-notified and on-the-spot inspections. Specifically, the study evaluated the use of Motivational Interviewing (MI), a psychological counselling method, by inspectors during their interactions with inspectees. The aim was to explore the extent to which MI training of inspectors was successful, and to evaluate the experience of inspectors using MI in their professional interactions with inspectees. The underlying assumption was that training in a psychological counselling method would be helpful for the inspectors (Steg & Vlek, 2009).

MI has been widely used with health behaviour problems, and has a solid research base with more than 200 randomised controlled studies, which in the main have shown significant low to moderate effect sizes in respect of, e.g., reducing or stopping problem drinking, stopping the use of illegal drugs and tobacco use, and completing a treatment program (Lundahl et al., 2010; Lundahl et al., 2013; Hettema, Steele & Miller, 2005). The positive effects of MI have contributed to its dissemination within health care but rarely beyond, barring a few exceptions, e.g., adopting clean drinking water practices (Thevos, Quick & Yanduli, 2000).

MI practice is focused on behaviour change. A short definition of MI is ‘a collaborative conversation style for strengthening a person’s own motivation and commitment to change’ (Miller & Rollnick, 2013). According to the MI model, it is assumed that people prefer to take their own decisions regarding matters that affect them, and that they may often take offence when their choices are questioned (Miller & Rollnick, 2013). MI counsellors are trained to interact in a manner that is empathic and collaborative. Information is shaped so that it is more likely to be requested and understood. When using MI, counsellors are required to evoke the client’s motivation and belief in the ability to change undesirable target behaviours into those that are desirable. This is because there is an established correlation between clients expressing reasons in favour of change during conversations, and the realisation of actual behaviour (Apodaca & Longabaugh, 2009).

One previous study has shown MI to be feasible to use in conversations with people about their environmental behaviour, and to increase pro-environmental verbal behaviours compared to controls (Klonek & Kauffeld, 2012). To our knowledge, this is the first study in which environmental protection inspectors have used MI. Based on social psychology and learning psychology theories, we hypothesise that MI may be a suitable tool for evoking long-term universal values; as such MI may offer untapped potential for more effective work within the environment (Larsson, 2011; Larsson, 2013). MI may be useful in evoking a broad range of reasons in favour of environmental preservation, such as forward-looking human values (e.g., a desire to preserve the environment for one‘s children and grandchildren), which may counteract the desire to keep short-term economic costs to a minimum. In the context of this study, we hypothesise that MI may be effective in reinforcing sustainable environmental behaviour among inspectees.

## Material and Methods

### Source of data

The present work was carried out as part of the research program: *Efficient environmental inspections and enforcement* (EMT: Effektiv miljötillsyn). Its aim was to analyse Swedish environmental inspections and enforcement from a variety of perspectives. This sub-study was conducted between September 2011 and October 2012 in four municipalities in different parts of Sweden. The municipalities invited to participate in the study were Östersund, a large town of 44,000 inhabitants, and Ale, Nybro, and Älmhult, which are small municipalities. The head of the Operational Inspection and Enforcement Authority for the Environment (OIEAE) in each municipality selected inspectors based on their willingness to participate in the study. In all, 40 inspectors, 2 heads of the OIEAE, and 3 secretaries participated. Table 1 shows age, gender and type of inspection (which type of target behaviour that the inspection targeted) within the four municipalities.

**Table 1:** Age, gender and inspection target behaviour under different regulations and codes for inspectors within four municipalities.

Municipality	All inspectors				Inspection target behaviour under the law and administration of		
	Men	Women	≤ 35 yrs	≥ 35 yrs	National food administration	National environmental protection agency	National board of housing, building and planning
Ale	0	8	1	7	4	4	
Nybro	6	4	6	4	2	8	
Älmhult	2	4	3	3	2	2	2
Östersund	8	8	7	9	5	11	
Total	16	24	17	23	13	25	2

The study was approved by the Regional Board of Ethics in Stockholm (2012/1:7). All inspectors were provided with oral and written information regarding the study. They were informed that their participation was voluntary and that no negative consequences would ensue, should they choose not to participate or withdraw later on. In turn, inspectors informed inspectees that they had received MI training, that they would like to audio record the conversations for use in the MI training, and that they could choose whether to participate and that, in the event of participation, they could withdraw later without any negative consequences.

### MI training

Prior to the start of the study, a pilot MI training protocol was developed and tested in a fifth municipality (Eksjö) with six inspectors plus the head of the OIEAE and the final MI training model was adjusted based on these experiences. The MI training was divided into six days, each day having a different theme (Table 2). A day contained three hours of theory and exercises, and three hours of feedback on the participants ‘audio-recorded live inspection conversations. Audio-recorded conversations and transcripts of recorded inspections were used as training material to help clarify MI terms, and to reinforce inspectors’ MI skills. The reason for splitting training into six separate days was to allow inspectors time to record live conversations between training days. During feedback sessions, the inspectors were divided into two groups of three to four members. MI skills were assessed by independent coders, who listened to the recorded conversations whereupon they described the skills in a protocol. The participants’ learning of the different MI skills were based on the protocols.

**Table 2:** Themes for each of the six days of MI-training.

Training day	MI–theme
DAY 1	*To listen and convey cooperation*
	– To understand the meaning of MI
	– To listen and engage in conversation
	– To convey cooperation and equality
	– To direct toward a target behaviour
DAY 2	*To strengthen reinforces toward positive behaviours*
	– To recognize, elicit, and strengthen change talk
	– To ask open-ended and exploring questions
DAY 3	*To exchange information and to understand the mechanisms of reinforcement*
	– To inform as a dialogue
	– To understand and implement positive and negative reinforcement
	– To convey listening by reflecting
DAY 4	*To make efforts to understand the perspectives of the other*
	– To use empathic listening
	– To convey that you are listening and trying to understand through reflections
	– To use MITI-coding as feedback
DAY 5	*To meet ambivalence and resistance*
	– To explore readiness to change and ambivalence
	– To meet and roll with resistance
	– To avoid MI-non-adherent utterances
DAY 6	*To use MI in daily inspection practice*
	– To summarize what has been gone through at the training
	– To form a personal plan for upholding MI-proficiency

The theory and practice element of MI training was offered to all inspectors in each of the four municipalities. In total, 40 inspectors participated in this part of the training. Eight inspectors did not take part in the feedback element of training because they did not carry out inspections. Thirty-two inspectors contributed audio-recorded conversations (17 inspectors under the regulation of the National Environmental Protection Agency, 13 under the Codes of the National Food Administration, and 2 under the National Board of Housing, Building, and Planning).

**Figure 1:**
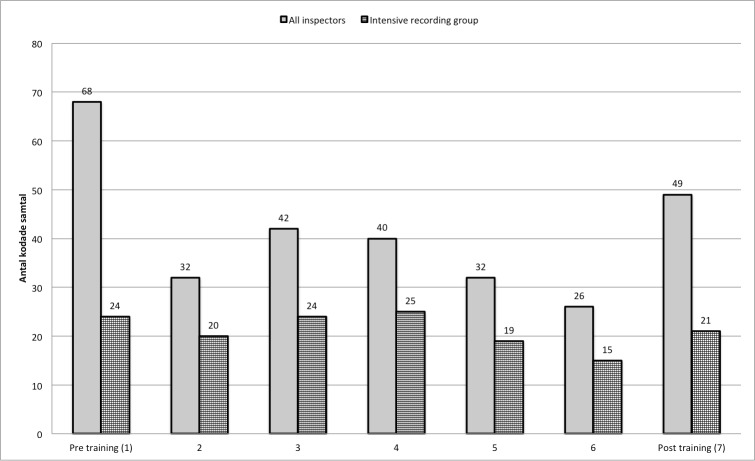
Number of coded conversations per training day for 32 inspectors and for 9 inspectors in the intense recording group.

In addition to their use as training material, the conversations recorded during the training period were also used to evaluate inspectors’ MI acquisition. Three conversations were chosen pre- and post-MI training in order to get an average estimate of the inspector conversation practice. During the MI training program, inspectors were asked to record one conversation prior to each training day. In order to have a sample of inspectors with accurate measures of MI competency that were relatively independent of the inspectee and inspection situation, two inspectors per municipality (three in one municipality) were asked to record three conversations prior to each training day. These nine inspectors formed a more intense recording group. In total, inspectors successfully recorded 76 percent of planned recordings (Table 3). Three coders coded all 289 conversations, of which 148 were from the intense recording group (Fig. 1).

**Table 3:** Number of planned and recorded conversations and per cent dropout (number) from planned recordings distributed for each municipality.

Municipality	All 32 inspectors, including 9 intense recording group members		
	Number of planned conversations	Number of recorded and coded conversations	Per cent dropout (numbers)
Ale	82	56	32 (26)
Nybro	94	67	29 (27)
Älmhult	85	57	33 (28)
Östersund	119	109	8 (10)
Total	380	289	24 (91)

### Data collecting of MI skills

All recorded conversations were evaluated for MI proficiency by professional coders at the Motivational Interviewing Coding Laboratory at Karolinska Institute (MIC Lab) in accordance with the Motivational Interviewing Treatment Code (MITI) (Moyers et al., 2007) using a Swedish translation of the MITI 3.1 (Forsberg, Forsberg & van Loo, 2008). The MITI evaluates a 20-min practice sample and has been used for providing feedback in MI training and for assessing MI integrity in research (Madson & Campbell, 2006). The MITI has proved discriminant validity for measuring change in an individual’s MI skills over time, and between individuals who had and had not received MI training (Forsberg et al., 2008). The MITI rating scheme rated practitioner verbal behaviours. Coding involved the scoring of five global variables; Direction, Empathy, and MI Spirit (comprising three sub-variables: Evocation, Collaboration and Autonomy Support), which were rated on a five-point Likert-type scale ranging from 1 (low) to 5 (high) and the counting of the frequency of seven verbal behaviours; Giving Information, MI Adherent and MI Non-Adherent statements, Questions (Closed and Open), and Reflections (Simple and Complex). Detailed instructions on how to rate the global variables and the behavior counts are provided in the MITI manual (Moyers et al., 2007).

The MIC Lab coders, who coded the material for the present study, had worked at the MIC Lab since 2009 and 2010 and had undergone a four week initial training, followed by three-hour training sessions every week, including regular inter-rater checks. The reliability of the three coders was calculated by random recoding of 10% of the 289 recordings coded during the study period. The reliability for the five global variables was estimated as the percentage agreement between the coders and as intra-class correlations (ICC), calculated in a two-way mixed model, with absolute agreement and reported as single measures. For the seven behaviour count variables, only the ICC was calculated (Table 4).

**Table 4:** Reliability between three coders estimated in a random sample (*n* = 28) of 289 recordings. Intra-class-correlations (ICC) are calculated as mixed models, with absolute agreement, and reported as single measures. Also, percentage agreement (numbers).

MITI global variables	Exact agreement for all three coders	One of the coders is one step difference from the other two coders	One of the coders is two steps difference from the other two coders	ICC
Empathy	50% (14)	43% (12)	7% (2)	0.35
Evocation	39% (11)	54% (15)	7% (2)	0.36
Collaboration	36% (10)	57% (16)	7% (2)	0.47
Autonomy	36% (10)	57% (16)	7% (2)	0.53
MI-spirit				0.67
Direction	46% (13)	46% (13)	7% (2)	0.21
Information				0.44
MI-adherent				0.52
MI-non-adherent				0.70
Closed questions				0.70
Open questions				0.76
Simple reflections				0.78
Complex reflections				0.22

The ICC for the global variable Empathy was poor according to Cicchetti (1994), who proposed that the significance of an ICC below .40 is poor, an ICC between .40 and .59 is fair, ICC between .60 and .74 is good, and an ICC between .75 and 1.00 is excellent. However, when inspecting the double coding of the Empathy variable in more detail as percent agreement between coders, 26 of 28 coded samples differed no more than one step on the scale. The poor ICC could be explained by the coders’ use of the five point scale (only three steps of the scale were used, because the conversations were of similar types in the selected sample). As shown in Table 4 the reliability on Direction, Collaboration, Autonomy support and Evocation had similar reliability as Empathy; all the global variables had adequate reliability and could be used in further analyses. The ICC for the behaviour counts; Closed questions, Open questions, Simple reflections and MI-non-adherent statements ranged from “good” to “excellent” (0.64–0.87) and the ICC for the MI-adherent statements variable was fair. All these behaviour counts could be used in analyses. However, the ICC was poor for Complex reflections and no further analyses with Complex reflections were carried out.

### Questionnaire about MI experiences

To follow the inspectors’ experience of using MI during their routine inspections and to gather feedback on the MI training, they were asked to fill in a questionnaire on each training day (Table 5). The questionnaire had five questions on a six-point scale, where six means very useful and one means not useful at all. Three of the questions related to the inspector’s experience of the training day and the different parts of it: (1) How would you rate the use of feedback on recorded conversations? (2) How would you rate the theory element of training? (3) How would you rate the practical exercises? The two other questions were: (4) How would you rate the training as a whole? (5) How likely are you to apply your new knowledge during your inspections?

**Table 5:** Inspector participation at six training days, submitted questionnaires and per cent questionnaire dropout (numbers).

Municipality	Number of inspectors	Per cent attendance at all six training days (number)	Submitted questionnaire	Per cent dropout (number)
Ale	8	60 (29)	23	21 (6)
Nybro	10	86 (52)	39	25 (13)
Älmhult	6	97 (35)	31	11 (4)
Östersund	16	100 (117)	101	14 (16)
All municipalities	40	233	195	16 (38)

### Inspection questionnaire at inspections

The inspector’s experience of the inspections during the yearlong period of training was monitored by an inspection questionnaire. The inspector filled in a web questionnaire (SurveyMonkey; www.surveymonkey.com) after each inspection. This questionnaire was linked to the inspector and to the date of the filing, so as to be able to relate each inspection to the MI training process. The inspection questionnaire was administered for three municipalities after the first training day and in one municipality prior to the first training. The questionnaire contained 10 statements on the inspector’s experience of the inspection on a 5-point scale (5 = “very well”; 4 = “corresponds somewhat”; 3 = “neither well or bad”; 2 = “not that well”; 1 =“very bad”):

1. At the beginning of the visit, the inspectee had a positive attitude to the inspection;
2. I consider that following the visit the inspectee had enough knowledge about how his own activity influences the environment and/or health;
3. I consider that the inspectee clearly demonstrated that he/she understood the information I wanted to convey during the inspection;
4. I consider that the inspectee has carried out the required measures/demands toward reducing his/her influence on the environment/ensuring food safety;
5. I consider that the inspectee will need to take steps toward reducing his/her influence on the environment/ensuring food safety;
6. I consider that the inspectee will take steps toward reducing his/her influence on the environment/ensuring food safety;
7. I consider that the inspectee conveyed his/her own reasons and motives to take the necessary action during the conversation;
8. I consider that the inspectee showed an interest in contributing to sustainable development of the environment;
9. I was satisfied with my own work during the inspection;
10. I consider that the inspection was a positive experience for the inspectee.

The inspection questionnaire also contained 8 statements about the inspection and the inspectee’s activity:

(1) Was the meeting considered to be an inspection (from the inspector’s perspective)? (2) Was this the first visit to this inspectee? (3) Type of supervised activity? (4) Was the investigation notified in advance? (5) Do the supervised activities require a permit or notification? (6) Type of inspectee? (7) Size of activity/number of employees (8) Have you or a colleague previously visited this inspectee during the research programme?

## Statistical Analyses

MITI global variables (Empathy, Evocation, Collaboration, Autonomy support and Direction) were coded on a five-point ordinal scale and were thus considered as non-parametric. Therefore, chi square analyses and Mann–Whitney U-tests were conducted (Table 6). The behavior counts (MI adherent and MI non-adherent utterances, Open and Closed questions, Simple and Complex reflections) were counts of verbal behaviors and considered as parametric (Table 7). The mean for each of the six training sessions and before and after MI-training were compared in a one way ANOVA analysis. All analyses were conducted using SPSS 22.0.

**Table 6:** MITI global variables for all recordings (*n* = 289). Number of recordings per scale value on five-point ordinal Likert scales per MI training occasion. Expected counts in parenthesis.

Recording MITI variable	Scale value	1st training	2nd	3rd	4th	5th	6th	After training
Empathy	1	19 (16.3)	14 (10.7)	17 (13)	12 (13)	11 (10.7)	4 (9.6)	7 (10.7)
	2	10 (10.7)	3 (7)	4 (8.5)	11 (8.5)	6 (7)	11 (6.3)	10 (7)
	>=3	0 (1.9)	2 (1.3)	2 (1.5)	0 (1.5)	2 (1.3)	2 (1.1)	2 (1.3)
		Chi-square (12) = 23.25, *p* = 0.026						
Evocation	1	24 (17.2)	12 (10.7)	19 (13.5)	13 (13.7)	8 (11.5)	5 (10.1)	7 (11.3)
	2	4 (9.4)	5 (5.8)	2 (7.5)	9 (7.5)	8 (6.2)	11 (5.5)	9 (6.2)
	>=3	1 (2.4)	1 (1.5)	2 (1.9)	2 (1.9)	3 (1.5)	1 (1.4)	3 (1.5)
		Chi-square (12) = 29.12, *p* = 0.001						
Collaboration	1	11 (7.4)	4 (4.8)	10 (5.9)	7 (5.9)	3 (4.8)	2 (4.3)	1 (4.8)
	2	16 (16.5)	10 (10.8)	8 (13.1)	14 (13.1)	12 (10.8)	11 (9.7)	14 (10.8)
	>=3	2 (5.1)	5 (3.3)	5 (4.0)	2 (3)	4 (3.3)	4 (3.0)	4 (3.3)
		Chi-square (12) = 18.01, *p* = .115						
Autonomy	1	6 (5.7)	4 (3.5)	8 (4.5)	7 (4.5)	2 (3.7)	2 (3.3)	0 (3.7)
	2	18 (14.7)	8 (9.1)	7 (11.7)	11 (11.7)	12 (9.6)	8 (8.6)	11 (9.6)
	>=3	5 (8.6)	6 (5.4)	8 (6.8)	5 (6.8)	5 (5.6)	7 (5.1)	8 (5.6)
		Chi-square (12)= 16.92, *p* = 0.153						
Direction	1	0 (0.4)	1 (0.2)	1 (0.3)	0 (0.3)	0 (1.1)	0 (0.2)	0 (0.3)
	2	3 (1.4)	1 (0.8)	2 (2)	1 (1.1)	0 (0.9)	0 (0.8)	0 (0.9)
	>=3	26 (27.2)	16 (16.9)	21 (22.6)	22 (21.6)	19 (17.9)	17 (16)	19 (17.9)
		Chi-square (12) = 10.94, *p* = 0.534						

**Table 7:** MITI behaviour count variables for all recordings (*n* = 289). Mean and (standard deviation/sd) per behaviour count variable in MITI and MI training occasion and after the MI training (*n* = inspectors per training occasion) were calculated.

Recording MITI variable		1st training	2nd	3rd	4th	5th	6th	After training
		(*n* = 29)	(*n* = 20)	(*n* = 25)	(*n* = 23)	(*n* = 19)	(*n* = 17)	(*n* = 19)
Information	mean (ds)	17.6 (6.0)	18.5 (8.1)	15.2 (8.1)	16.4 (6.0)	16.5 (8.0)	13.6 (3.4)	13.2 (3.6)
MI-adherent	mean (ds)	0.1 (0.2)	0.1 (0.3)	0.1 (0.3)	0.2 (0.3)	0.3 (0.4)	0.3 (0.5)	0.3 (0.4)
MI-non-adherent	mean (ds)	3.8 (3.6)	2.7 (3.3)	3.5 (3.2)	3.3 (2.5)	2.4 (2.1)	2.7 (2.6)	2.0 (1.4)
Closed questions	mean (ds)	12.8 (6.8)	14.2 (7.2)	15.0 (7.5)	13.7 (6.4)	12.2 (5.6)	13.0 (4.4)	15.3 (4.7)
Open questions	mean (ds)	3.7 (3.6)	4.0 (4.0)	5.1 (4.0)	4.0 (3.8)	4.1 (2.9)	4.3 (2.7)	4.0 (3.3)
Open question/question ratio	mean (ds)	.28 (.64)	.30 (.72)	.40 (.93)	.38 (.45)	.42 (.35)	.47 (.58)	.31 (.42)
Simple reflections	mean (ds)	6.4 (3.4)	5.7 (3.9)	7.6 (6.7)	7.5 (4.9)	6.7 (4.7)	6.6 (1.5)	6.3 (4.5)
Reflection/Question ratio	mean (ds)	.46 (0.29)	.34 (.19)	.40 (.34)	.56 (.39)	.51 (.36)	.52 (.27)	.43 (.27)

## Results and Discussion

### MITI measures reflecting MI-skills

The inspectors significantly increased their competence in the Empathy global variable, calculated with chi-sqr (df 12) = 23.25; *p* = 0.026 (two-sided) during the training period (Table 6). There were no differences in Empathy development between municipalities. Empathy scores have been shown to positively correlate with outcomes in health behaviour studies (Moyers & Miller, 2013). A question for future research is whether an increase in Empathy scores will have impact on inspectee pro-environmental behaviours. However, current expert opinion is that the inspectors’ Empathy scores should be at a higher competency level (Moyers et al., 2007) in order to positively influence inspectees’ pro-environmental behaviours.

The relatively low Empathy scores and MI competency as measured by MITI during the course of training might be explained by inaccurate MI training. The MI competency level reached by inspectors was too low, necessitating measures to increase MI skill acquisition by inspectors. The attained low level of MI competence might also be explained by the different inspection types selected for monitoring. In the selected sample, inspections targeted problematic behaviours, which were regulated by different legislative regimes. It was therefore difficult to know in which part of the conversations it was appropriate to use MI. Among the selected recordings there were many conversations in which MI was not appropriate to use e.g., where it was not clear which inspectee behaviour was targeted, or where inspectees were not in a position to make decisions to change current environmental behaviours.

More close data analyses revealed differences in Empathy scores related to type of inspection. On a Mann–Whitney U-test, the inspectors of the food administration demonstrated significantly higher scores in Empathy compared to environmental- and health protection inspectors (*p* = 0.039). *T*-tests also showed significantly higher scores for all the behaviour count variables among the food administration inspectors compared to the others (all *p*-values were less than 0.05). These differences in MI practice between different types of inspection may indicate that MI may be easier or more appropriate to use in certain inspections than in others. This is a question for future research.

Inspectors significantly increased their competence in the global variable, Evocation (chi-sqr (df 12) = 29.12; *p* = 0.001; two-sided) but in Collaboration (chi-sqr (12 df) = 18.01, *p* = 0.115) and in the Autonomy support variable (chi-square (12) = 16.92, *p* = 0.153) there were no significant changes. On average, the inspectors’ scores on the Empathy and Evocation variables were lower than those on the Collaboration and Autonomy support variables. This suggests that these two variables measured skills that were more difficult for inspectors to learn.

In the MITI behaviour counts the number of utterances providing information had decreased, but not significantly (Table 7). The decrease was interesting since it may reflect that inspectors were giving inspectees’ points of view more room in the conversations. Another interesting finding was that the number of MI-non-adherent utterances had decreased by 50% among inspectors (not significant). Inspectors of the food administration decreased the MI-non-adherent utterances from just over 5 per conversation pre-training to just over 2 post-training (tending toward significance; *t* = 1.74, df = 18, *p* < 0.1). Clinical MI trials, have found an inverse relation between MI-non-adherent utterances and behaviour change (Apodaca & Longabaugh, 2009). Therefore, a future research question is whether a decrease in inspectors’ MI-non-adherent utterances relates to an increase in inspectees’ pro-environmental behaviours. According to a chi-square test only MI- adherent statements developed significantly over time (*χ*2 = 40.06, df = 24, *p* = 0.021). This could be seen in Table 7 as a higher spread at the 6th and 7th occasion than at earlier occasions.

### Questionnaire about MI experiences

In questions 1–3 of the questionnaire about the inspectors’ MI experience, inspectors considered each training day to be useful—approximately 5 on a 6-point scale, where 6 means very useful and 1 means not useful at all. There were no changes in these judgements over the yearlong training period or between municipalities. When the inspectors’ answers about the usefulness of the training day were analysed in detail, the theory sessions scored 5/6, and the feedback on the recorded conversations scored between 4.5/6 and 5/6. None of these judgements changed over time. In all, the inspectors had a positive evaluation of the training.

A crucial question in the questionnaire was whether the inspector considered MI to be useful when carrying out inspections. The responses showed that the inspectors judged MI to be useful in inspections, approximately 5 on the 6-point scale. These evaluations did not change over time. The responses indicate that inspectors in four different municipalities considered MI to be useful in the daily inspection routine throughout the 10–13 month long study period. MI’s usefulness to inspectors seemed to be maintained after the novelty value of taking part in MI training had decreased.

### Questionnaire at inspections

The inspection questionnaire had been filled in before the training only in one of the municipalities, Östersund. The other municipalities started to fill in the web questionnaire after training day 1. Seven inspectors did not file any questionnaires two inspectors completed the most questionnaires, with 116 questionnaires and 95 completed respectively. These two inspectors accounted for a little more than 40% of the questionnaire material. However, their satisfaction did not increase over time; their satisfaction with their own inspection practice was constantly high, and therefore did not contribute to an increase in satisfaction for the group. On the question whether inspectors were satisfied with their work during the inspections, the proportion who had answered “very well” increased from 26.1% to 51.3% while the part who had answered “very bad” or “not that well” or “neither well or bad” decreased from 31.5% to 3.2%. The results indicate that the inspectors’ satisfaction with their inspections increased over the study period, which may be related to acquisition of MI competency.

The inspectors estimated how inspectees viewed the inspections by the question: “I consider that the inspection was a positive experience for the inspectee”. In the responses to this question, inspectors more often (37%; 19/51) considered that their inspections had been a positive experience for the inspectee after MI-training compared with before (not significant), which may indicate that by using MI, inspectors had been able to improve their interactions with interviewees.

### Limitations and strengths of the study

A strength of the study was that the inspectors used MI during a long time period (10–13 months), during which their competency in MI and experience of MI were continuously monitored. The evaluation period allows us to be reasonably confident in the inspectors’ evaluation of MI as well as how well MI competency was attained. A limitation of the study was that municipalities were not randomized, thus the results cannot be generalized to all Swedish municipalities. However, the four selected municipalities represented different parts of Sweden, and included large as well as small municipalities, which increases the representativeness of the results. Another limitation was a large dropout rate in returning inspection questionnaires among inspectors, which means that the results pertaining to these questionnaires should be interpreted with caution. Some inspectors also failed to audio record inspection conversations. However, these missing recordings may not have impaired the results, since the MI competence of the inspectors appeared to be stable for the group as a whole. The recordings in both the intense recording group of inspectors (who made three recordings prior to each training day) and the remaining inspectors (who had one recording prior to each training day) reached the same levels of MI competency. Another strength of the study was that the assessment of inspectors’ MI competency was reliably monitored throughout the long study period, except for the MITI variable, complex reflections.

## Conclusion

Inspectors considered MI useful in environmental inspection and enforcement interactions with inspectees, which indicates that MI facilitated the work of inspectors. Future research should examine whether MI does affect inspectee environmental behaviour. It may also be important to explore more efficient MI training and to adapt the training to the type of inspections for which it is appropriate to use MI.

## Additional Information and Declarations

### Data Deposition

The following information was supplied regarding the deposition of related data: Raw data is kept at the MIC Lab research group at the Department of Clinical Neuroscience, Karolinska Institutet, Sweden and will be made available upon email request: Lars Forsberg, Lars.Forsberg.3@ki.se.
